# Differential motif enrichment analysis of paired ChIP-seq experiments

**DOI:** 10.1186/1471-2164-15-752

**Published:** 2014-09-02

**Authors:** Tom Lesluyes, James Johnson, Philip Machanick, Timothy L Bailey

**Affiliations:** Institute for Molecular Bioscience, The University of Queensland, 306 Carmody Road, 4072 Brisbane, Australia; Department of Computer Science, Rhodes University, Drosty Road, 6140 Grahamstown, Eastern Cape Province South Africa

**Keywords:** Comparative ChIP-seq analysis, Constrained differential motif enrichment analysis, MCF10A-ER-Src cells, ChIP-seq, Regulation of transcription, Gene expression

## Abstract

**Background:**

Motif enrichment analysis of transcription factor ChIP-seq data can help identify transcription factors that cooperate or compete. Previously, little attention has been given to *comparative* motif enrichment analysis of pairs of ChIP-seq experiments, where the binding of the same transcription factor is assayed under different conditions. Such comparative analysis could potentially identify the distinct regulatory partners/competitors of the assayed transcription factor under different conditions or at different stages of development.

**Results:**

We describe a new methodology for identifying sequence motifs that are *differentially* enriched in one set of DNA or RNA sequences relative to another set, and apply it to paired ChIP-seq experiments. We show that, using paired ChIP-seq data for a *single* transcription factor, differential motif enrichment analysis identifies *all* the known key transcription factors involved in the transformation of non-cancerous immortalized breast cells (MCF10A-ER-Src cells) into cancer stem cells whereas non-differential motif enrichment analysis does not. We also show that differential motif enrichment analysis identifies regulatory motifs that are significantly enriched at constrained locations within the bound promoters, and that these motifs are not identified by non-differential motif enrichment analysis. Our methodology differs from other approaches in that it leverages both *comparative* enrichment and *positional* enrichment of motifs in ChIP-seq peak regions or in the promoters of genes bound by the transcription factor.

**Conclusions:**

We show that differential motif enrichment analysis of paired ChIP-seq experiments offers biological insights not available from non-differential analysis. In contrast to previous approaches, our method detects motifs that are enriched in a *constrained region* in one set of sequences, but not enriched in the same region in the comparative set. We have enhanced the web-based CentriMo algorithm to allow it to perform the constrained differential motif enrichment analysis described in this paper, and CentriMo’s on-line interface (http://meme.ebi.edu.au) provides dozens of databases of DNA- and RNA-binding motifs from a full range of organisms. All data and output files presented here are available at http://research.imb.uq.edu.au/t.bailey/supplementary_data/Lesluyes2014.

**Electronic supplementary material:**

The online version of this article (doi:10.1186/1471-2164-15-752) contains supplementary material, which is available to authorized users.

## Background

Sequence motifs in DNA and RNA molecules are key players in the regulation of gene expression. Proteins and RNA molecules bind to these motifs in a sequence-specific way to control transcription and subsequent sequestration or degradation of messenger RNA (mRNA). High-throughput sequencing technology has given us access to genome-wide measurements of mRNA levels (e.g., RNA-seq) as well as protein-DNA (e.g., ChIP-seq) or protein-RNA (e.g., CLIP-seq) interactions [1]. Advances in protein-binding microarrays and high-throughput variants of SELEX have recently been used to produce large compendia of both DNA [2–4] and RNA motifs [5]. These two threads of technological advancement provide the necessary inputs for very productive analyses of the regulatory roles of sequence motifs associated with particular DNA- or RNA-binding molecules.

In this paper we describe a methodology for detecting sequence motifs that are enriched in one set of sequences relative to another set. This is called differential motif enrichment analysis (DMEA), and is a type of motif enrichment analysis [6]. Motif enrichment analysis differs from *de novo* motif discovery in that a set of known, well-characterized motifs are part of the input to motif enrichment analysis. Motif enrichment analysis has two major strengths relative to motif discovery. Firstly, because the motifs come from curated motif databases, the identities of the biological molecules that bind them are known. Secondly, restricting attention to the curated set of motifs increases statistical power, allowing more subtle motif enrichments to be detected. This latter advantage is simply a consequence of the huge number of possible sequence motifs that *de novo* motif discovery must consider.

The DMEA approach we describe also takes advantage of positional information, in contrast to other motif enrichment analysis approaches, such as AME [6], which measure enrichment over a whole genomic region. For example, ChIP-seq and CLIP-seq technologies identify the (approximate) loci where a protein interacts with DNA or RNA, respectively. The resolution of the loci depends on the technology and is approximately 50 bp for ChIP-seq [7]. DMEA can leverage this fact by focusing on motifs that are enriched in the central 100 bp portion relative to the flanks, of genomic regions identified by ChIP-seq. This is the approach taken by the original CentriMo algorithm [8], and is still available in the enhanced version of that algorithm that we describe here. A fortunate side-effect of using positional information in this way is that the flanking regions provide a built-in negative control for the statistical test of motif enrichment.

Positional information can also be leveraged by DMEA when motifs occur at preferred locations anywhere (not just centrally) within the input sequences. Examples of where this is useful include promoters for expressed genes aligned on their start of transcription (TSS) or ChIP-seq regions aligned on the best match to the known motif of the binding protein. In the former case, regulatory motifs frequently occur at preferred locations relative to the TSS (e.g., the TATA-box around 30 bp upstream of mammalian TSSs [9]). In the latter case, co-regulatory proteins frequently bind in particular configurations [10]. In the new version of the CentriMo algorithm described here, we allow the user to relax the requirement that the enriched region be centrally located. This allows CentriMo to be applicable in a wider range of scenarios.

The major contribution of this paper is to describe and illustrate differential local motif enrichment analysis. We show that DMEA can identify biologically relevant motifs that are relatively enriched in one set of ChIP-seq peaks compared to another. Importantly, in the example we study here, these relevant motifs are *not* detected without the use of differential analysis. In addition, we apply differential enrichment analysis to two sets of promoters, bound or unbound by a particular transcription factor, and detect a number of motifs for physiologically relevant motifs. Our analyses are based on published ChIP-seq data in transformed and untransformed versions of an immortalized breast cell line, but the approach is completely general and can yield biological insights in many experimental settings, as we describe in the Results and discussion section.

## Results and discussion

### Finding differentially enriched motifs in paired ChIP-seq experiments

Differential motif enrichment analysis can be used to analyze two ChIP-seq experiments for the *same* TF. One objective of such an analysis is to determine if the ChIP-ed TF changes co-factors between the two experiments. Given two sets of ChIP-seq peak regions for TF *X* from experiments *A* and *B*, known motifs differentially enriched in set *A* relative to set *Bmay* indicate that *X* is co-regulating some of its targets in conjunction with different TFs in the two experiments. Hints as to the identities of the co-factors are provided by the annotation associated with the known, differentially enriched motifs.

One caveat to this type of analysis is that the observed differential enrichment of motifs may be an artifact of the relative efficiency of the two ChIP-seq experiments. For example, if experiment *A* was more successful than *B* at predicting the *actual* bound sites of TF *X*, the peak regions in set *A* may be more enriched for some co-factor motifs even though those motifs are not truly differentially enriched in the true populations of binding sites of TF *X* in the two experiments. It would therefore be incorrect to claim that enrichment of a motif in set *A* relative to set *B* was evidence of differential co-factor use. On the other hand, it would be valid to make this claim for motifs enriched in set *B* relative to set *A*.

Fortunately, it is easy to determine which of two ChIP-seq experiments for a TF *X* was more successful using the enhanced CentriMo algorithm. We simply run CentriMo and look for differential enrichment of the known DNA-binding motif for the ChIP-ed TF in set *A* relative to set *B*. If the known DNA-binding motif for the ChIP-ed TF is significantly differentially enriched (*E*-value of the Fisher exact test <0.05, henceforth the “Fisher *E*-value”), it is unsafe to use set *B* as the control. Conversely, if the Fisher *E*-value of the known motif for TF *X* reported is much larger than 1, it is safe to use set *B* as the control. This can be confirmed by running CentriMo with the roles of sets *A* and *B* swapped.

**Figure 1:**
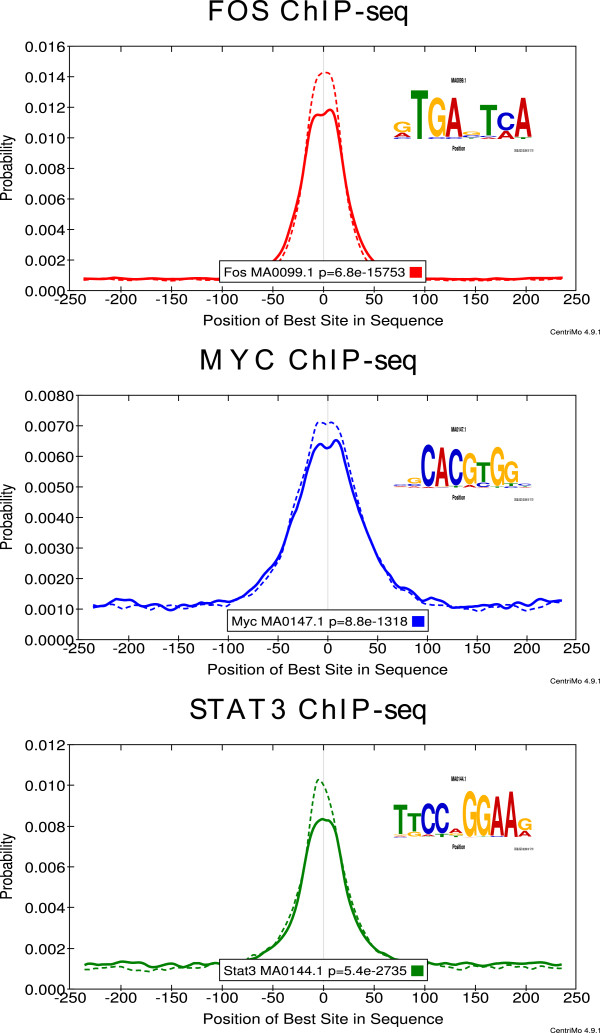
**The known motif for the ChIP-ed motif is more enriched in ChIP-seq peak regions from** ***untreated*** **MCF10A-ER-Src cells.** The CentriMo plots show the distribution of a known motif for the ChIP-ed TF in FOS, MYC and STAT3 ChIP-seq peak regions (top-to-bottom). Solid (dotted) curves show the positional distribution of the known motif in the tamoxifen-treated (untreated) cell ChIP-seq peak regions. Tamoxifen treatment time is 4 hours except in the STAT3 ChIP-seq experiment where it is 12 hours. JASPAR motif names and IDs and the *p*-value of the motif’s central enrichment in the treated cell peaks is shown in the legend of each plot.

We performed this analysis on the pairs of ChIP-seq experiments for tamoxifen-treated and EtOH-treated (“untreated”) MCF10A-ER-Src (Table 1). Treatment of these cells with tamoxifen has been shown to lead to self-renewing mammospheres that contain cancer stem cells [11]. In all these experimental pairs, it happens that the the known motif for the TF is significantly relatively enriched in the “untreated” cells. This is apparent from the highly significant Fisher *E*-value when the peak regions for the untreated cells (set *B*) are used as the “treatment” set in CentriMo’s input. Conversely, when the treated cell data (set *A*) are given as the “treatment” set to CentriMo, the Fisher *E*-value is always its maximum possible value (884, the number of motifs given as input to CentriMo). The CentriMo site probability curves for the known motif for three “A vs. B” cases from Table 1 are shown in Figure 1. The results in Table 1 and Figure 1 make it clear that it is only safe to make inferences about differentially enriched motifs in the MCF10A-ER-Src ChIP-seq data if we use the untreated (EtOH) cells as the control, not vice-versa. Therefore, in what follows, we use CentriMo only to look for known motifs that are relatively enriched in the centers of ChIP-seq peak regions from tamoxifen-treated cells compared with untreated cells.

**Table 1:** **Relative enrichment of the motif for the ChIP-ed TF in tamoxifen-treated and untreated MCF10A-ER-Src cells**

					*Fisher E-value*
*ChIP*	*A*	*B*	*Motif*	*A vs. B*	*B vs. A*
FOS	Tam 4 hr	EtOH	Fos	884	**1.24e-87**
FOS	Tam 12 hr	EtOH	Fos	884	4.07e-59
FOS	Tam 36 hr	EtOH	Fos	884	1.24e-06
MYC	Tam 4 hr	EtOH	Myc	884	**4.95e-05**
STAT3	Tam 12 hr	EtOH	Stat3	884	**2.74e-11**
STAT3	Tam 36 hr	EtOH	Stat3	884	1.15e-10

The table columns show the name of the ChIP-ed transcription factor (“ChIP”), the names of the two ChIP-seq peak region sets (columns “A” and “B”), the name of the known (JASPAR) motif for the ChIP-ed TF (“Motif”) and the Fisher *E*-value (adjusted for 884 known motifs) reported by CentriMo when the first-named peak region set is used as the “treatment” and the second as the “control”. CentriMo site probability curves for the cases in bold font and the JASPAR IDs of the known motifs are given in Figure 1.

For each of the three ChIP-ed factors—FOS, MYC and STAT3—CentriMo differential motif enrichment analysis identifies one or both of the *other* two factors as the most significantly enriched known motif after tamoxifen treatment (Table 2). The effect sizes (odds ratios, see Additional file 1: Tables S2–S7) for these six relative enrichments are large, ranging from 1.18 to 1.41, showing that the enriched motifs for the secondary factors are at least 18% more likely to occur near the center of the ChIP-seq peak in the treated cells. For example, with tamoxifen treatment of 12 or 36 hours, the most relatively enriched motif in the FOS ChIP-seq peaks is a STAT motif, and vice-versa (Table 2, first line). A STAT motif is also the most differentially enriched in the peaks from cells treated for 4 hours. In the MYC ChIP-seq experiment, CentriMo reports that the three most differentially enriched motifs in the treated cell peaks are FOS and STAT motifs (odds ratios from 1.19 to 1.20). All these differential motif enrichments are highly significant statistically (Fisher *E*-value<10^-7^) and suggest that FOS, MYC and STAT3 begin binding in close proximity to each other at many genomic loci in MCF10A-ER-Src cells after treatment with tamoxifen.

**Table 2:** **Relatively enriched motifs in tamoxifen-treated vs. untreated MCF10A-ER-Src cells**

FOS					
**4 hr**		**12 hr**		**36 hr**	
***Motif***	***E-value***	***Motif***	***E-value***	***Motif***	***E-value***
STAT1	**5.7e-22**	STAT1	**1.6e-24**	STAT1	**1.5e-17**
Stat3	**7.2e-18**	Stat3	**3.3e-21**	Stat3	**1.4e-05**
FLI1_full	**1.3e-08**	FEV	**1.1e-14**	ATF4_DBD	**2.2e-05**
FEV_DBD	**1.9e-08**	ETS1_DBD	**1.1e-13**	NFIL3_DBD	**7.4e-05**
ETV4_DBD	**3.1e-08**	ELK3_DBD	**1.3e-13**	HLF_full	**0.00015**
ELK1_DBD	**5.7e-08**	FLI1_full	**1.4e-13**	ETV1_DBD	**0.00032**
FLI1_DBD	**5.8e-08**	ERG_DBD	**1.6e-13**	ETV4_DBD	**0.00037**
ERG_DBD	**8.1e-08**	ERG_full	**1.6e-13**	HLF	**0.00048**
ELK3_DBD	**8.8e-08**	ELK1_DBD	**2.2e-13**	ELK3_DBD	**0.00063**
ETS1_full	**1.1e-07**	RELA	**3.3e-13**	ETV6_full_2	**0.0011**
**MYC**		**STAT3**			
**4 hr**		**12 hr**		**36 hr**	
***Motif***	***E-value***	***Motif***	***E-value***	***Motif***	***E-value***
Fos	**5.7e-17**	Fos	**1.8e-42**	Fos	**1.7e-32**
AP1	**3.4e-16**	AP1	**9.7e-33**	AP1	**2.2e-22**
STAT1	**1.1e-08**	JDP2_DBD	**1.5e-21**	JDP2_full	**3.4e-20**
Stat3	**0.0027**	JDP2_full	**1.1e-20**	JDP2_DBD	**1.2e-19**
RELA	**0.0085**	Jdp2_DBD	**6.4e-17**	Jdp2_DBD	**5.3e-18**
NF-kappaB	**0.0088**	NFE2_DBD	**3e-14**	NFE2_DBD	**1.2e-10**
NFE2L1::MafG	0.065	NFE2L1::MafG	**6.8e-09**	NFE2L1::MafG	**8e-09**
CEBPA	0.11	NFE2L2	**9.7e-05**	MEOX2_DBD	**0.0077**
FEV	0.28	MAFF_DBD	0.27	NFE2L2	**0.0078**
REL	0.49	Pax2	0.3	MEOX1_full	0.097

The table shows the ten most differentially enriched motifs in ChIP-seq peaks for the given ChIP-ed TF (top lines) in MCF10A-ER-Src cells treated with tamoxifen for the given time (second lines) compared with untreated cells. The name of the JASPAR or Jolma *et al.* [3] motif and its Fisher *E*-value as computed by CentriMo are given and each column in the table is sorted by *E*-value. Significant (***≤***0.05) *E*-values are shown in bold font. All enriched motifs have odds ratios at least 1.09.

**Figure 2:**
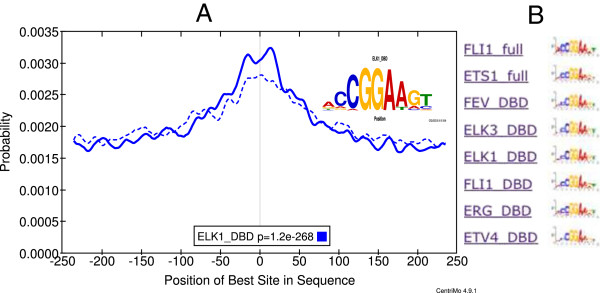
**ELK1 and other ETS-factor motifs are relatively enriched in FOS ChIP-seq peaks from tamoxifen treated MCF10A-ER-Src cells.** Panel **A** shows the central enrichment of the ELK1_DBD motif (from Jolma *et al.*[3]) in the treated-cell (solid line) and untreated-cell (dotted line) FOS ChIP-seq peaks. Treatment was for 4 hours. Panel **B** shows the logos of the eight most differentially enriched ETS-factor motifs.

This CentriMo differential motif analysis also identifies motifs for several other TFs with known roles in MCF10A cells, suggesting that they may be bound by co-factors of FOS, MYC and STAT3 (Table 2). Possible co-factors that become more active upon tamoxifen treatment include ELK1, ATF4, and NF- *κ*B. ELK1 is known to regulate MCF10A breast epithelial cell migration [12]. The CentriMo site distribution plot for the ELK1 motif and the logos for it and the other similar motifs (mostly ETS factors) are shown in Figure 2. ATF4 forms heterodimers with both FOS and c-Jun [13], two members of the various AP1 complexes whose roles in MCF10A cells are now being explored [14]. The role of NF- *κ*B in tamoxifen treated MCF10A-ER-Src cells is well established. NF- *κ*B responds to the inflammatory response triggered by the transient presence of Src and leads to activation of STAT3, as reported by the group which produced the ChIP-seq datasets we use in this analysis [11].

Perhaps the most interesting motifs are those that are enriched in ChIP-seq peaks from tamoxifen-treated cells but *not* enriched before treatment. We can easily restrict the analysis to such motifs using CentriMo’s interactive HTML output, which allows filtering on various features including motif significance in either set of peaks. The nature of the association of binding by FOS, MYC, STAT3 and NF- *κ*B becomes clearer when we restrict the analysis to motifs that are not significantly enriched in un-treated MCF10A-Er-Src cells (Table 3, see Additional file 1: Tables S8–S13 for effect sizes). In FOS peaks, the relative enrichment of NF- *κ*B motifs is not significant after four hours of tamoxifen treatment (Fisher *E*-value=6.7), but becomes highly significant (Fisher *E*-value<10^-8^) after 12 hours of treatment. This association between FOS binding and NF- *κ*B motifs then disappears after 36 hours of treatment. By contrast, NF- *κ*B motifs are significantly differentially enriched in MYC ChIP-seq peaks after only four hours of tamoxifen treatment, but they are never differentially enriched in STAT3 peaks after treatment. The analysis presented in Table 3 also shows that CTCF motifs are differentially enriched in FOS peaks at the same treatment time point (12 hours) as NF- *κ*B motifs, and not differentially enriched in peaks for the other two TFs (MYC and STAT3). As in the previous analysis (presented in Table 2), CentriMo specifically identifies differential enrichment of motifs in the families of STAT3 and FOS in each others bound regions after treatment, suggesting that they regulate an overlapping set of targets in tamoxifen treated MCF10A-ER-Src cells. The new analysis in Table 3 highlights the transient role of NF- *κ*B in co-regulating the targets of FOS and MYC.

**Table 3:** **Motifs that are** ***not*** **enriched in untreated MCF10A-ER-Src cells but that** ***are*** **enriched in tamoxifen-treated cells**

FOS					
**4 hr**		**12 hr**		**36 hr**	
***Motif***	***E-value***	***Motif***	***E-value***	***Motif***	***E-value***
STAT1	**5.7e-22**	STAT1	**1.6e-24**	STAT1	**1.5e-17**
SPI1	**0.003**	REL	**2.7e-09**	BSX_DBD	0.8
CTCF	3.1	NF-kappaB	**7.4e-09**	UNCX_DBD	7.3
NFKB2_DBD	6.7	NFKB2_DBD	**2.2e-05**	PDX1_DBD_2	8.8
SPIB	27	NFKB1	**8.5e-05**	Dbp_DBD	17
RXR::RAR_DR5	159	ETS1	**0.00057**	NKX6-2_full	28
SP1	159	CTCF	**0.0065**	HNF1A_full	45
INSM1	177	SPIB	0.8	POU6F2_full	48
NFKB1	239	Spic_DBD	177	Dlx2_DBD	50
RARG_full_3	557	SPI1	212	Lhx8_DBD_2	50
**MYC**		**STAT3**			
**4 hr**		**12 hr**		**36 hr**	
***Motif***	***E-value***	***Motif***	***E-value***	***Motif***	***E-value***
STAT1	**1.1e-08**	AP1	**9.7e-33**	MEOX2_DBD	**0.0077**
NF-kappaB	**0.0088**	Hoxa2_DBD	42	DLX5_FL	0.11
NFKB1	1.2	SPIB_DBD	53	En2_DBD	0.13
NFKB1_DBD	9.7	POU1F1_DBD	239	POU1F1_DBD	0.22
NFKB2_DBD	9.7	VAX2_DBD	265	Meox2_DBD	0.57
EN1_full_2	12	En2_DBD	309	Hoxa2_DBD	0.7
NR2E1_full	15	MZF1_1-4	398	VAX1_DBD	1.6
TEAD1_full	23	ZIC3_full	415	EMX2_DBD	2.4
MEOX2_DBD	33	Nkx6-1_DBD	424	HOXB5_DBD	2.5
MZF1_1-4	36	HMBOX1_DBD	442	EVX2_DBD	4.5

The table shows the most differentially enriched motifs in ChIP-seq peaks in tamoxifen treated cells where the motif is not significantly enriched (*E*-value ***≥***1) in the untreated MCF10A-ER-Src cells. The first two lines show the ChIP-ed TF and the tamoxifen treatment time, respectively. The name of the JASPAR or Jolma *et al.* [3] motif and the significance of its differential enrichment (Fisher *E*-value) as computed by CentriMo are given and each column in the table is sorted by *E*-value. Significant (***≤***0.05) *E*-values are shown in bold font.

### The benefit of differential motif enrichment analysis in paired ChIP-seq experiments

As we have seen, the most highly differentially enriched motifs in the paired ChIP-seq experiments are extremely relevant to the biology of MCF10A cells. However, if we look at the central enrichment of the FOS-, MYC-, STAT- and NF- *κ*B-family motifs in the tamoxifen-treated cells, rather than at their differential enrichment, they are *not* among the most highly enriched. (This is conveniently done with CentriMo by choosing to sort by *E*-value rather than Fisher *E*-value using a drop-down menu in the CentriMo output.) For example, in the FOS ChIP-seq experiments, all MYC, STAT and NF- *κ*B motifs rank far down the list of 884 known motifs in terms of central enrichment (Table 4). Although a STAT-family motif (STAT1) is the most *differentially* enriched motif (Table 2 and Table 3), non-differential enrichment places all STAT-family motifs at rank 112 or below in the three tamoxifen-treated FOS ChIP-seq datasets (Table 4). Non-differential motif enrichment analysis thus does not make clear the important biological role of STAT3 in tamoxifen-treated MCF10A-ER-Src cells. The same is true for the role of NF- *κ*B, as motifs for NF- *κ*B rank far down the list in the non-differential enrichment analysis of FOS ChIP-seq peaks (rank ≥ 282, Table 4), whereas an NF- *κ*B motif is ranked third and fourth in the differential motif enrichment analysis of cells treated with tamoxifen for 4 hr or 12 hr (Table 3).

**Table 4:** **Central enrichment of STAT, FOS, MYC and NF-** ***κ*** **B motifs in ChIP-seq peaks in tamoxifen-treated MCF10A-ER-Src cells**

FOS					
**4 hr**		**12 hr**		**36 hr**	
***Motif***	***E-value***	***Motif***	***E-value***	***Motif***	***E-value***
JDP2_full	1	JDP2_full	1	JDP2_full	1
Fos	4	Fos	4	Fos	4
Mycn	125	Stat3	112	Mycn	117
Stat3	130	Mycn	122	Stat3	136
NFKB1_DBD	322	NFKB2_DBD	282	NFKB1_DBD	402
**MYC**		**STAT3**			
**4 hr**		**12 hr**		**36 hr**	
***Motif***	***E-value***	***Motif***	***E-value***	***Motif***	***E-value***
JDP2_full	1	STAT1	1	STAT1	1
Mycn	3	Fos	6	Fos	6
Fos	11	NF-kappaB	79	NF-kappaB	110
Stat3	181	Mycn	213	Mycn	221
NFKB2_DBD	317				

The table shows the name and rank (out of 884) of the most significantly enriched motif from each of the four TF families in the peaks of the ChIP-ed TF (top lines) in MCF10A-ER-Src cells treated with tamoxifen for the given time (second lines). The top-ranking motif is also shown even if it is not from one of the four TF families. Rank is based on the CentriMo (non-differential) central enrichment *E*-value.

The benefit of differential motif enrichment analysis is also seen in the MYC and STAT3 ChIP-seq experiments. Differential analysis of paired MYC ChIP-seq experiments places FOS, STAT and NF- *κ*B at the top of the list of 884 known motifs (Table 2), but non-differential analysis only highlights FOS motifs (Fos rank =11, Table 4). In the tamoxifen-treated cell MYC ChIP-seq experiment, STAT- and NF- *κ*B-family motifs rank below 180 out of 884 motifs. In the case of the STAT3 experiments, both differential and non-differential enrichment rank FOS motifs near the top of the list (Table 2 and Table 4), but differential analysis also ranks an NF- *κ*B motif at position 14 in the 12 hr treated cells (NFKB2_DBD, data not shown).

**Figure 3:**
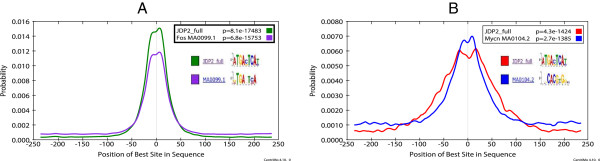
**Top non-differentially enriched motifs in ChIP-seq peak regions from tamoxifen-treated MCF10A-ER-Src cells.** The CentriMo plots show the distribution of the given motifs in the **(A)** FOS and **(B)** MYC ChIP-seq peak regions from MCF10A-ER-Src cells after 4 hr treatment with tamoxifen. The motif names and IDs and the *p*-value of the motif’s central enrichment in the ChIP-seq peaks is shown in the legend of each plot, and the motif logos are shown below the legend.

Non-differential central motif enrichment analysis continues to be useful for studying the DNA-binding affinity of the ChIP-ed TF. In all six tamoxifen-treated cell ChIP-seq experiments, a known motif from the ChIP-ed TF’s family ranks near the top of the list (Fos, Mycn and STAT1 motifs in FOS, MYC and STAT3 experiments, respectively, Table 4). In the three FOS experiments and the MYC experiment, the JDP2_full motif, which is highly similar to the Fos motif, ranks first. In the case of the FOS ChIP-seq peaks, this may indicate that the JDP2_full motif is may more faithfully represent the DNA-binding affinity of FOS than the JASPAR Fos motif does (Figure 3A). It is more surprising that the JDP2_full motif is more significantly centrally enriched than any MYC-family motif in the MYC ChIP-seq peaks. However, the enrichment of the Mycn motif in the MYC peaks is actually more spatially confined (Figure 3B). The regions of maximal central enrichment for the JDP2_full and Mycn motifs are 148 bp and 99 bp wide respectively (data not shown). Thus, despite the lower *p*-value of the JDP2_full motif, the non-differential central motif enrichment analysis allows correct identification of the Mycn motif as most similar to the primary DNA-binding motif of the ChIP-ed TF, MYC.

### Differential local enrichment of motifs in bound and unbound promoters

CentriMo can be used to perform *local* motif enrichment analysis in regions aligned at a genomic landmark such as the transcription start site (TSS) of each of a set of genes. Other useful genomic landmarks such as translation start sites, intron-exon boundaries and polyadenylation sites can also be used to align genomic regions to be analyzed by CentriMo. In addition, if two sets of regions are provided to CentriMo, it can also be used to perform *differential* local motif enrichment analysis.

Using the same ChIP-seq data from MCF10A-ER-Src as before, we performed differential local motif enrichment analysis comparing promoter regions *near* FOS, MYC or STAT3 binding sites (“bound promoters”) with promoter regions *distal* from any binding site (“unbound promoters”). To do this, for each ChIP-seq experiment we split the annotated human TSSs (hg19, UCSC Genes) into two sets depending on whether they were within 1000 bp of a declared ChIP-seq peak or not. We then created two input files containing the 500 bp regions centered on the TSSs of these promoters and used them as input CentriMo. We will refer to the first set as “FOS-bound” promoters and the second as “FOS-unbound” promoters, etc.

In untreated MCF10A-ER-Src cells the motifs for FOS, MYC and STAT3 are *not* the most locally enriched motifs in the 500 bp regions centered on TSSs near ChIP-seq peaks for the respective transcription factors (Table 5). In terms of local enrichment, the highest rank for a motif from the ChIP-ed TF’s family is 125 (out of 884, STAT3).

**Table 5:** **Local motif enrichment in bound and unbound promoters in** ***untreated*** **MCF10A-ER-Src cells**

	FOS		MYC		STAT3	
	*Motif*	*Rank*	*Motif*	*Rank*	*Motif*	*Rank*
*Local Enrichment: Proximal TSSs*	SP4_full	1	SP4_full	1	KLF14_DBD	1
	Fos	288	Mycn	365	STAT1	125
	Mycn	323	STAT1	482	Mycn	505
	STAT1	874	Fos	587	Fos	541
*Differential Enrichment: Proximal vs. Distal TSSs*	JDP2_DBD	1	NFYA	1	Stat3	1
	Fos	11	Myc	63	MYC::MAX	795
	Mycn	33	Fos	532	Fos	829
	STAT1	857	STAT1	559		

The table summarizes local motif enrichment around TSSs proximal (within 1000 bp) or distal to the nearest peak in FOS, MYC and STAT3 ChIP-seq experiments in *untreated* MCF10A-ER-Src cells. For each ChIP-ed factor, we report the name (in the JASPAR+Jolma compendium) and rank (out of 884) of the most significantly enriched motif from each of the three ChIP-ed TF families. in *untreated* MCF10A-ER-Src cells. Rank is based on either the local enrichment in bound promoters (“Proximal TSSs”) or the differential local enrichment between bound and unbound promoters (“Proximal vs. Distal TSSs”). The top-ranking motif is also shown even if it is not from on of the three ChIP-ed TF families. The numbers of proximal (distal) TSSs are FOS: 4503 (44548); MYC: 11192 (37859); and STAT3: 2261 (46790).

On the other hand, motifs from the ChIP-ed TFs family show strong differential local motif enrichment when comparing bound and unbound promoters. Notably, a motif for STAT3 ranks first in terms of differential local enrichment in bound vs. unbound promoter regions (Table 5), compared with a best rank of 125 in terms of local motif enrichment in bound promoter regions. Motifs from the FOS and MYC families also have significantly higher ranks (Fos: 11 vs. 288 and Myc/Mycn: 63 vs 365) in promoters bound by FOS and MYC, respectively.

We note also that the motif for NF-YA (JASPAR motif NFYA) is highly differentially enriched enrichment in promoters bound by MYC (Table 5, rank 1, Fisher *E*-value <10^-38^), but less so in promoters bound by FOS (rank 24, Fisher *E*-value 0.01, data not shown). Recent work in different cell lines (K562, GM12878, HeLa S3) indicated an association between MYC and NF-Y at promoters, and a strong association between NF-Y and FOS at loci lacking the AP-1 motif [15].

Selecting the individual motifs for display in the CentriMo interactive report for FOS bound/unbound promoters shows the JASPAR AP1 motif is present in 34% of the bound promoters, and the JASPAR NFYA motif in 22%. Choosing both motifs for display causes CentriMo to report the intersection size as well—8%—which is barely above what would be expected by chance if the presence of the two motifs were uncorrelated (34*%*·22*%*=7.48*%*).

Thus, CentriMo reveals that motifs for NF-Y and AP-1 do not seem to be associated at FOS-bound promoters in MCF10A-ER-Src cells, as was previously shown in other cell lines [15].

**Figure 4:**
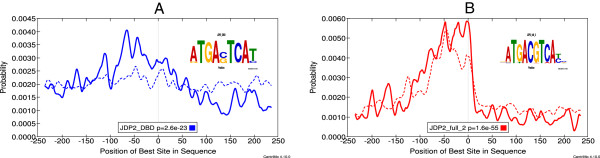
**TRE and CRE motifs near promoters bound by FOS in** ***untreated*** **MCF10A-ER-Src cells.** The CentriMo plots show the distribution of a known motif for the ChIP-ed TF around TSSs that are near (≤1000 bp) a FOS ChIP-seq peaks (solid curve) or more distal (dotted curve). The plots are centered on the TSS. Panel **A** shows the motif (a CRE motif) with most significant *differential* local enrichment (Fisher *E*-value =1.4·10^-10^); panel **B** shows the third most locally enriched motif (a TRE motif) around FOS-bound promoters (non-differential *E*-value =1.4·10^-52^. Motif IDs and the *p*-value of the motif’s local enrichment near the FOS-bound promoters is shown in the legend of each plot.

The CentriMo analysis of the local differential motif enrichment in the FOS ChIP-seq dataset is particularly interesting (Figure 4). The most differentially enriched motif in bound vs. unbound promoters is JDP2_DBD, a cAMP response-element (CRE) motif for the DNA-binding domain (DBD) of the Jun dimerization protein2 (JDP2). This motif is essentially the same as that of other FOS family members, The differential local enrichment of this motif is highly significant (Fisher *E*-value <10^-9^, Figure 4A), but it is *not* locally enriched in the unbound promoters (*E*-value = 884). However, JDP2 can also bind a TPA-response element (TRE), and this motif ranks third in terms of *local* enrichment (Figure 4B), but is *not* significantly differentially enriched according to the CentriMo analysis (Fisher *E*-value = 3.1). As seen in the logos in Figure 4, the TRE and CRE motifs differ only in the distance between the palindromic ATGA/TCAT half-sites. The CentriMo analysis thus reveals that *all* human promoters are locally enriched for TRE motifs, but FOS-bound promoters in MCF10A-ER-Src cells are *relatively* enriched for the closely related CRE motif typical of FOS binding in the 100 bp region upstream of the TSS.

**Figure 5:**
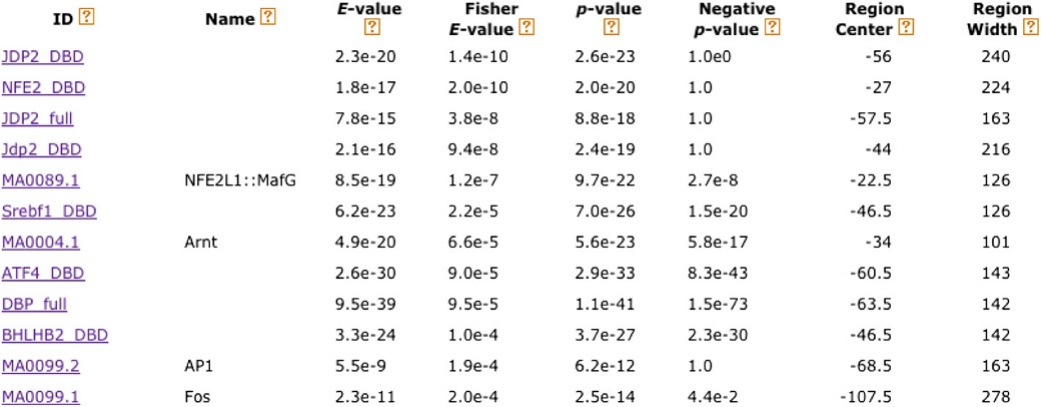
**Local differential enrichment of motifs in FOS-bound vs. unbound promoters.** A (partial) screenshot of the CentriMo interactive output using FOS-bound and unbound promoters shows the twelve most locally differentially enriched motifs in the JASPAR+Jolma compendium. For each motif, the table shows its ID and name in the compendium, the local enrichment in the bound promoters (“*E*-value”), the differential enrichment (“Fisher *E*-value”) in the bound vs. unbound promoters, the (unadjusted) significance of the local enrichment in the bound promoters (“*p*-value”), the (unadjusted) significance of the local enrichment in the unbound promoters (“Negative *p*-value”), and the coordinates of the region of maximum enrichment in the bound promoters (“Region center” and “Region width”).

Local differential motif enrichment analysis of FOS-bound and unbound promoters also highlights several motifs that may be of interest in the study of breast cancer. When we sort the CentriMo output by differential enrichment (Fisher *E*-value, Figure 5), among the top twelve motifs are seven motifs similar to the Fos-family consensus (TGANTCA), including the first five motifs. A search of the literature reveals that three of the five remaining motifs belong to proteins with possible links to breast cancer: include Srebf1_DBD, Arnt and DBP_full. Srebp1 (sterol regulatory element-binding protein-1), also known as Srebf1, may be involved in breast cancer stem-like cell survival [16], as was shown using MCF10A and MCF10AT cells [16]. Lipogenesis, of which Srebp1 is a master regulator, may play a critical role in early breast carcinogenesis, and increased expression of lipogenic enzymes seems to correlate with increased risk of development of breast cancer [17]. Arnt has been shown to have a role in estrogen receptor-negative breast cancer [18]. A role in the regulation of BRCA2 has been demonstrated for DBP (vitamin D-binding protein) [19]. We did not find any known link to breast cancer for the remaining two motifs, ATF4_DBD and BHLHB2_DBD. The high proportion of biologically interesting motifs among the most significant differentially enriched motifs in FOS-bound promoters demonstrates the potential value of this use of CentriMo.

## Conclusions

We have shown that *differential* local motif enrichment analysis can yield insights beyond those available from motif analysis of a single dataset. Using this new feature of the CentriMo algorithm, we showed that the differential analysis of ChIP-seq peaks for a single transcription factor under two different cellular conditions identifies several other transcription factors with pivotal roles in the distinguishing the two cellular states. In particular, CentriMo differential analysis of ChIP-seq of just FOS in transformed and untransformed MCF10A-ER-Src cells ranks motifs from the STAT and NF- *κ*B families first and second in terms of statistical significance (Table 3, FOS 12 hr column), whereas non-differential analysis ranks them 112 and 282 out of 884 motifs (Table 4). Given that STAT3 and NF- *κ*B have been shown to be the key transcription factors in the positive feedback loop that maintains the transformed state of MCF10A-ER-Src after removal of the tamoxifen stimulus [11], this result shows the potential of differential local motif analysis of paired ChIP-seq experiments to identify important candidate transcription factors for further investigation.

We have also demonstrated the utility of differential local motif analysis is also useful for analyzing signals near genomic landmarks such as TSSs. One such application is the comparative motif analysis of promoters bound our unbound by a particular transcription factor. Using some of the same ChIP-seq data for FOS as above, CentriMo differential local motif analysis revealed the a probable association between binding of FOS and a MYC family member, most likely MYC at promoters in MCF10A-ER-Src cells. The same analysis also highlighted the widespread presence of TRE motifs in human promoters and the lack of the related (one extra base-pair) CRE motif in promoters not bound by FOS in MCF10A-ER-Src cells.

One can imagine many other scenarios where differential motif analysis would be useful. We studied paired ChIP-seq experiments before and after a treatment. The same types of analysis could be applied to pairs of ChIP-seq experiments from different cell or tissue types, or from the same tissue at different developmental stages. CentriMo could also be used with RNA-binding protein (RBP) motifs to study paired CLIP-seq (or equivalent) datasets. The CentriMo website currently provides one large compendium of RBP motifs [5] and users can also upload their own sets of motifs. Our local motif enrichment analysis focused on promoters near to or distal from ChIP-seq peaks, but one could also examine sets of promoters grouped using other criteria (e.g., expressed vs. not expressed in a given tissue). In short, differential motif analysis can be applied whenever paired sets of genomic regions or RNA molecules may harbor encoded signals, regulatory or otherwise.

A single CentriMo differential motif enrichment analysis can be used in many different ways. The CentriMo report is highly interactive and allows the user to sort the results by non-differential enrichment, differential enrichment, position of enrichment, number of motif matches, and many other criteria. The user can also filter the results by non-differential or differential enrichment significance, the size of the enriched region or enrichment in the control dataset. The user also has full control over what information to display via check boxes on the report. The positional distribution of motif matches of user-chosen motifs is plotted and can be panned and zoomed interactively, and the plot can be in terms of either match position or distance from the sequence center. The CentriMo report also provides for creation of publication quality figures from the distribution plots.

The value of all forms of motif enrichment analysis, including the differential local analysis presented here, depends to a large extent on the availability of high-quality, annotated motif databases. Fortunately a large and growing number of such databases are now available for both transcription factors and RNA-binding proteins. These databases, which are made available for use with CentriMo via its website, are based on a number of technologies including ChIP-seq, high-throughput SELEX, and protein binding microarrays. The complementary strengths and weaknesses of these different experimental technologies makes it advisable to repeat motif enrichment analyses using motif databases based on different technologies.

Among the databases made available by the CentriMo website are DNA-binding motif databases, including comprehensive databases of vertebrate motifs, specialized databases for particular organisms, and RNA-binding motif databases. For vertebrate ChIP-seq data, the comprehensive databases (e.g., “All Vertebrates”) will be the most useful since they contain the largest spectrum of known motifs. For data from non-vertebrate organisms, using the most relevant JASPAR specialized database (e.g., “JASPAR CORE (2014) fungi”) may be appropriate. Users may also input custom databases of motifs.

## Methods

### The CentriMo algorithm

CentriMo is a web-based visualization and statistical analysis tool for performing several types of motif enrichment analysis using one or two sets of equal-length DNA or RNA regions and sets of annotated motifs. It is partly based on an earlier, much simpler algorithm with the same name [8], but has vastly greater capabilities. While the original algorithm could only detect motifs enriched in the centers of a single set of sequences using a fixed motif-score threshold, CentriMo finds motifs enriched in any sub-region of the sequences and measures their relative enrichment in a comparative set of sequences, as well as automatically finding the optimal motif-score threshold. Unlike the earlier algorithm, CentriMo features interactive output for plotting the positional distribution of one or more of the significantly enriched motifs, displays the motif logos and allows the user to create custom publication-quality images of the plots. The user can also choose which motifs to display and view the sizes of the intersection and union sets of sequences containing the motif in the sub-region of enrichment. The user can also extract the sequence identifiers of the set of sequences that contain all of the chosen, enriched motifs. The CentriMo output also allows the user to sort and filter the results by motif enrichment in the primary set of sequences, differential enrichment, location or size of the constrained sub-region of enrichment of each motif, or by one of a large number of other characteristics of the data.

The CentriMo user provides one or two sets of DNA or RNAsequences, and selects (or provides) a set of DNA or RNA motifs in position weight matrix (PWM) format. CentriMo scores [20] each sequence in both the primary and control datasets using the PWM for a given motif, storing the position of the best match. By default CentriMo ignores sequences without a match above a user-defined score threshold, but it can also choose the threshold individually for each motif in order to maximize its statistical significance in the primary dataset. The locations of the best matches to the motif in the primary dataset are then subjected to a statistical analysis to determine local sub-regions of enrichment. A second, differential statistical analysis is then applied using both the primary and control sequence datasets to determine the relative enrichment of the motif in each of these sub-regions of local enrichment.

The statistical analysis of local motif enrichment performed by CentriMo is based on the binomial test. The “enrichment *p*-value” of a sub-region is the probability that the number of best matches to the motif falling in the sub-region would be at least as large as observed given a uniform prior. In other words, if *M* and *N* are the number of possible starting positions for the motif in the sub-region and in a whole single sequence, respectively, the uniform prior suggests that a random best site would fall in the sub-region with probability . CentriMo uses this *r* as the probability of a success in a single Bernoulli trial. If there are *s* best matches in the sub-region and *S* sequences (with best matches), the enrichment *p*-value is the probability of ≥*s* successes in *S* trials each with probability of success *r*.

Enrichment *p*-values are adjusted for multiple tests before being reported by CentriMo. Under the conservative assumption that sub-regions are independent, the probability that at least one sub-region out of *n* has *p*-value≤*p*^′^ is *p*=1-(1-*p*^′^)^*n*^. CentriMo reports *p*, the “adjusted *p*-value” of the motif, as well as its *E*-*value*—the expected number of motifs with adjusted *p*-value ≤*p*. The *E*-value is computed by multiplying *p* by the number of input motifs.

The original CentriMo algorithm limited its consideration to *centered* sub-regions in the equal-sized sequences (hence the name of the old algorithm). CentriMo now can test *all* possible sub-regions for enrichment. This increases the number of multiple tests performed, which reduces the significance level reported after *p*-value adjustment. However, when a motif’s sub-region of enrichment is not symmetrical around the centers of the sequence (e.g., when analyzing promoters), the new “local enrichment” mode of CentriMo can be more sensitive than the default “central enrichment” mode. Because CentriMo tests sub-regions of all possible widths and placements in local enrichment mode, its time complexity increases to quadratic in sequence length compared, with linear time complexity in central enrichment mode.

A single motif may be significantly enriched in multiple sub-regions. To avoid redundancy in its output, CentriMo uses a greedy strategy ensure that only *non-overlapping* significant sub-regions are reported. To do this, it sorts significant sub-regions by increasing enrichment *p*-value and outputs each sub-region in turn as long as it does not overlap an already-reported sub-region for the given motif. To maintain compatibility with the original algorithm, the user may limit the CentriMo search to central sub-regions only, in which case CentriMo reports the single sub-region with the most significant enrichment *p*-value for each motif.

CentriMo’s approach to differential local motif enrichment analysis only reports sub-regions where the motif is significantly enriched in the *primary* set of sequences. Once all the significant sub-regions in the primary sequences have been determined for a given motif, CentriMo then applies the Fisher exact test [21] to each sub-region to determine the significance of the relative enrichment of the motif in that region in the primary and control sequence sets. The test is computed on the 2 ×2 contingency table with rows labeled “success/failure” and columns labeled “primary/control”. The entries in the table are the number best matches in the sub-region (“success”) or outside the sub-region (“failure”) in the two sets of input sequences, respectively. The *p*-value of the test is the sum of the probability of all the 2 ×2 contingency tables with hypergeometric probabilities at least as small as that of the observed table. CentriMo adjusts the *p*-values for multiple tests (sub-regions) as described above, and also reports the “Fisher *E*-value” computed as the product of the adjusted Fisher *p*-value and the number of motifs in the CentriMo’s input.

We also considered other approaches to differential motif enrichment analysis, such as the obvious one of choosing the region that optimizes the Fisher exact test on the numbers of best matches in the region in the two sets of sequences. However, these approaches tended to identify extremely narrow sub-regions of enrichment that had no apparent biological interpretation (data not shown). In addition, these alternative approaches are far more computationally expensive.

### Data

All our motif enrichment analyses are based on ChIP-seq peaks from tamoxifen-treated and untreated MCF10A-ER-Src cells produced by the Struhl lab at Harvard University [1]. The data includes ChIP-seq experiments using antibodies against FOS, STAT3 and c-Myc. We downloaded this data in narrowPeak format from the UCSC Genome browser ENCODE website (http://genome.ucsc.edu/ENCODE). The datasets we use were created by the ENCODE Analysis Working Group (AWG) using a uniform analysis pipeline. The filenames of the datasets are given in Table 6.

**Table 6:** **MCF10A-ER-Src ChIP-seq data files**

*TF*	*Treatment*	*File name*
FOS	36 hr EtOH	wgEncodeAwgTfbsSydhMcf10aesCfosEtoh01HvdUniPk.narrowPeak.gz
	4 hr tamoxifen	wgEncodeAwgTfbsSydhMcf10aesCfosTam14hHvdUniPk.narrowPeak.gz
	12 hr tamoxifen	wgEncodeAwgTfbsSydhMcf10aesCfosTam112hHvdUniPk.narrowPeak.gz
	36 hr tamoxifen	wgEncodeAwgTfbsSydhMcf10aesCfosTamHvdUniPk.narrowPeak.gz
MYC	36 hr EtOH	wgEncodeAwgTfbsSydhMcf10aesCmycEtoh01HvdUniPk.narrowPeak.gz
	4 hr tamoxifen	wgEncodeAwgTfbsSydhMcf10aesCmycTam14hHvdUniPk.narrowPeak.gz
STAT3	36 hr EtOH	wgEncodeAwgTfbsSydhMcf10aesStat3Etoh01UniPk.narrowPeak.gz
	12 hr tamoxifen	wgEncodeAwgTfbsSydhMcf10aesStat3Tam112hHvdUniPk.narrowPeak.gz
	36 hr tamoxifen	wgEncodeAwgTfbsSydhMcf10aesStat3TamUniPk.narrowPeak.gz

The table shows the name of the file (“File Name”) on the UCSC ENCODE website (http://genome.ucsc.edu/ENCODE) containing the ChIP-seq peaks for the given transcription factor (“TF”) assayed in MCF10A-ER-Src cells after treatment with 0.01% ethanol or 1 *μ*m tamoxifen for the stated time (“Treatment”).

For our paired ChIP-seq experiment analyses we created genomic sequence sets corresponding to the ChIP-seq regions by extracting the 500 bp region around the center of each ChIP-seq region specified in a narrowPeak file in Table 6. We followed the sequence extraction protocol described in [22]. The genomic regions are from UCSC human genome assembly hg19 and are repeat-masked. For our promoter analyses, we used the set of human (hg19) transcription start sites denoted “UCSC Genes” available via the UCSC Genome Table Browser (http://genome.ucsc.edu/cgi-bin/hgTables).

For all our motif enrichment analyses we used a compendium of 884 motifs (“JASPAR+Jolma compendium”) that we created by combining all vertebrate motifs from JASPAR [23] with all the SELEX-based motifs from [3]. These two sets of motifs are available on the MEME Suite website in the MEME motif format required by CentriMo (http://meme.ebi.edu.au/meme/meme-download.html).

### Motif ranks

To find the highest-ranking FOS-, STAT-, MYC- or NF- *κ*B-family motifs in CentriMo output we searched the centrimo.txt output file for the most significant motif whose name contained the letters ‘fos’, ‘stat’, ‘myc’ or ‘kappa’, respectively. Significance was based on the log-adjusted *p*-value of the motif in the positive dataset (column 6 in centrimo.txt).

## Availability of supporting data

All input data and CentriMo output files described in this article are available at: http://research.imb.uq.edu.au/t.bailey/supplementary_data/Lesluyes2014.

## Electronic supplementary material

Additional file 1: **This file contains a detailed description of ChIP-seq datasets used in the paper, and a study comparing standard motif enrichment analysis with local motif enrichment analysis.** (PDF 969 KB)
