# Supplementary material for: Differential motif enrichment analysis of paired ChIP-seq experiments
Source: BMC Genomics. 2014 Sep 2;15(1):752. doi: 10.1186/1471-2164-15-752 (PMC4167127; doi:10.1186/1471-2164-15-752)
Supplement: Supplementary file 1 — Additional file 1: This file contains a detailed description of ChIP-seq datasets used in the paper, and a study comparing standard motif enrichment analysis with local motif enrichment analysis. (PDF 969 KB) [file 12864_2014_6441_MOESM1_ESM.pdf]

Additional File 1 for Differential motif enrichment analysis of paired ChIP-seq experiments

Tom Lesluyes, James Johnson, Philip Machanick and Timothy L. Bailey

Data

All files in Additional file 1: Table S1 are from `ftp://hgdownload.cse.ucsc.edu/goldenPath/hg19/encodeDCC/wgEncodeAwgTfbsUniform/`, and are from the ENCODE September 2012 Uniform data set (ENCODE Consortium, 2012). Note that while there is significant variation in the number of peaks (binding regions identified by ChIP-seq), the number of regions in each case is large enough to be representative of binding, provided there is no systematic bias in the ChIP-seq experiment. In the case of FOS, the "untreated" (EtOH) example has about the same number of peaks as one of the "treated" examples (36 hr tamoxifen), so it is unlikely that depth of coverage is a factor in the results.

Standard *vs.* comparative local motif enrichment analysis of a leukaemia cell line

To further illustrate the benefit of central motif enrichment analysis over standard motif enrichment analysis when used to detect differences in motif usage between two sets of sequences, we study two TATA-binding protein (TBP) ChIP-seq datasets. TBP is a control gene used in minimal residual disease (MRD) testing for leukaemia (van der Velden *et al.*, 2003). We compare TBP binding specificity in a cell line derived from healthy blood (GM12878) with a leukaemia cell line (K562) using ENCODE ChIP-seq data. To illustrate the difference between central motif enrichment analysis in differential mode and conventional MEA, we also use the AME algorithm on this example (McLeay and Bailey, 2010). AME can be run using Fisher's exact test to compare primary and control sequence sets, and we use this mode so the comparison with CentriMo in differential mode uses a similar statistical model (though comparing different things: CentriMo measures enrichment in a region, centred or otherwise, whereas AME compares enrichment over the whole width of each sequence).

Four out of the top five motifs identified by CentriMo in terms of differential enrichment (Fisher E-value $< 10^{-7}$) are for interferon proteins. CentriMo shows that the regions bound by TBP in GM12878 cells are centrally enriched for interferon-binding motifs, whereas TBP-bound regions in K562 (leukaemia) cells are not. This result is interesting in light of the fact that interferon is used in the treatment of leukaemia. Perhaps treatment of leukaemia cells with interferon activates some of the genes controlled by the TBP-bound set of regions that contain the enriched interferon motif. At any rate, the differential central motif enrichment analysis performed by CentriMo clearly indicates interferon as a molecule of interest in understanding the difference between normal cells and leukaemia cells. Although this result is not surprising in itself, it demonstrates the value of differential motif enrichment analysis using CentriMo.

The primary advantage of (differential) central motif enrichment is seen by the reduction in the number of statistically significant motifs found. In this example, CentriMo finds 23 motifs with significant differential local enrichment (Fisher E-value < 0.05). This is 2.6% of the total compendium of 884 motifs. By contrast, AME in differential mode finds 53 motifs (6% of the compendium) with enrichment p-value 10^{-100} (Bonferroni corrected), and 581 motifs (over 65% of the total compendium) with a corrected p-value < 0.05. The reporting of such a large number of differentially enriched motifs by AME makes identifying interesting candidate proteins much more difficult.

Like CentriMo, AME identifies interferon motifs as among the most differentially enriched in normal vs. leukaemia cells. However, the CentriMo re-

TF	Treatment	File Name	peaks
FOS	36 hr EtOH	wgEncodeAwgTfbsSydhMcf10aesCfosEtoh01HvdUniPk.narrowPeak.gz	67918
	4 hr tamoxifen	wgEncodeAwgTfbsSydhMcf10aesCfosTam14hHvdUniPk.narrowPeak.gz	86321
	12 hr tamoxifen	wgEncodeAwgTfbsSydhMcf10aesCfosTam112hHvdUniPk.narrowPeak.gz	92350
	36 hr tamoxifen	wgEncodeAwgTfbsSydhMcf10aesCfosTamHvdUniPk.narrowPeak.gz	70460
MYC	36 hr EtOH	wgEncodeAwgTfbsSydhMcf10aesCmycEtoh01HvdUniPk.narrowPeak.gz	35140
	4 hr tamoxifen	wgEncodeAwgTfbsSydhMcf10aesCmycTam14hHvdUniPk.narrowPeak.gz	25703
STAT3	36 hr EtOH	wgEncodeAwgTfbsSydhMcf10aesStat3Etoh01UniPk.narrowPeak.gz	12334
	12 hr tamoxifen	wgEncodeAwgTfbsSydhMcf10aesStat3Tam112hHvdUniPk.narrowPeak.gz	39861
	36 hr tamoxifen	wgEncodeAwgTfbsSydhMcf10aesStat3TamUniPk.narrowPeak.gz	44430
TBP	none	wgEncodeAwgTfbsSydhK562TbpIggmusUniPk.narrowPeak.gz	17529
	none	wgEncodeAwgTfbsSydhGm12878TbpIggmusUniPk.narrowPeak.gz	14892

Table S1: **MCF10A-ER-Src ChIP-seq data files.** The table shows the name of the file ("File Name") on the UCSC ENCODE website (`http://genome.ucsc.edu/ENCODE`) containing the ChIP-seq peaks for the given transcription factor ("TF") assayed in MCF10A-ER-Src cells after treatment with 0.01% ethanol or 1 μm tamoxifen for the state time ("Treatment"). The number of peaks (binding regions identified by ChIP-seq) may differ slightly from the number of loci in the original file as the count given here is after we eliminate duplicates. The last two rows are additional data only reported in this additional file.

sults provide provide an additional criterion for filtering motifs less likely to be of biological interest. Using the "negative p-value" filter, which measures the significance of central enrichment in the controt (K562) data, the user can see that all the significant interferon-family motifs have a p-value of 1.0 for the leukaemia (K562) cell line, and a significant E-value in the healthy (GM12878) cell line, ranging from 10^{-31} to 10^{-5}. In the AME results, while the interferon TFs dominate the most enriched end of the list, the much longer list of significantly enriched motifs makes it harder to isolate out those of most interest.

The CentriMo results also provide a richer form of comparison between two data sets because we can not only determine statistical enrichment but examine the distribution graph. In the case of interferons, it is known that interferon-based treatments can cause remission of chronic myeloid leukaemia (Greiner *et al.*, 2003) and since K562 is a chronic myeloid leukaemia cell line, it is useful to determine whether interferon-related TFs are differentially enriched when comparing K562 to GM12878.

Additional file 1: Figure S1 illustrates the top 4 interferon-family motifs (IRF) found by CentriMo to be differentially enriched in the healthy (GM12878) cell line versus the leukaemia (K562) cell line. The shape of the distribution indicates that these TFs are likely to bind in close proximity to the ChIP-ed TF, but not at a fixed spacing.

Additional file 1: Figure S2 illustrates how CentriMo finds a relatively small number of enriched motifs, and how those with significant p-value in the primary set with a p-value of 1 in the control set stand out. Compare with Additional file 1: Figure S3, where the first 15 motifs (all there is space for on the page) all have very low p-values for enrichment in the primary set.

A **B**

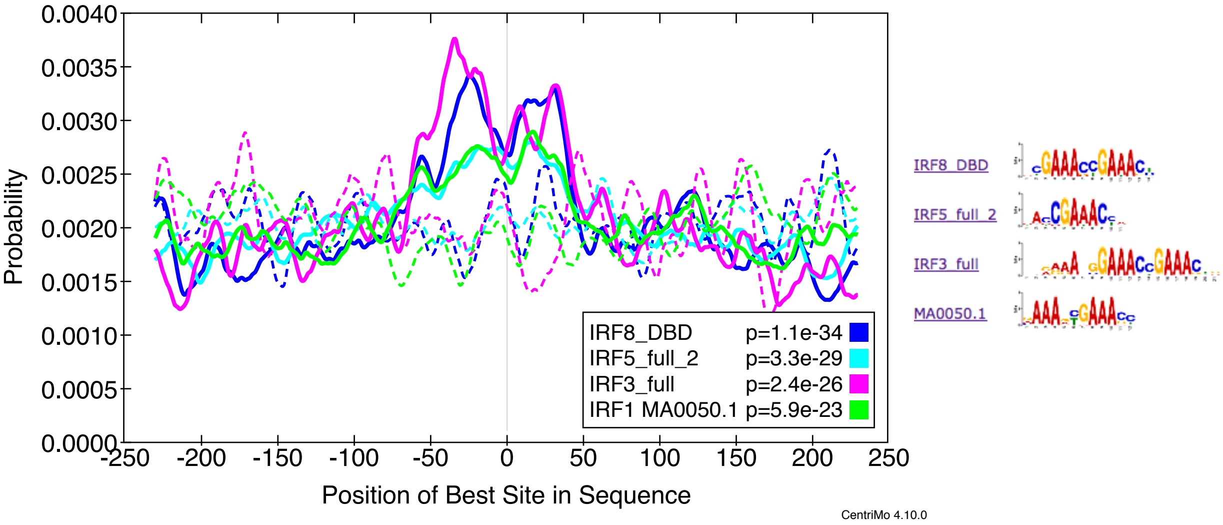

Figure S1: **Top 4 IRF motifs that are relatively enriched in TBP ChIP-seq TBP motif enrichment.** Panel A shows the central enrichment of th 4 most enriched IRF motif from Jolma *et al.* (2013) (first three motifs) and JASPAR (last motif) in the healthy cell line (solid line) and leukaemia cell line (dotted line) TBP ChIP-seq peaks. Panel B shows logos of the graphed motifs.

ID	Name	*E*-value	Fisher *E*-value	*p*-value	Negative *p*-value	Region Width	Region Matches	Negative Region Matches
MA0050.1	IRF1	5.3e-20	9.7e-16	5.9e-23	1.0	121	1960	1037
IRF5_full_2		2.9e-26	1.1e-11	3.3e-29	1.0	130	2419	1660
MA0137.1	STAT1	1.5e-35	5.8e-11	1.7e-38	1.0	121	1706	912
IRF8_DBD		9.8e-32	1.3e-8	1.1e-34	1.0	83	890	348
IRF3_full		2.2e-23	3.6e-8	2.4e-26	1.0	126	778	339
PRDM1_full		6.2e-17	2.5e-6	7.0e-20	1.0	118	1752	1111
SPIC_full		5.5e-28	2.0e-5	6.2e-31	6.3e-1	125	3239	2668
IRF7_DBD		6.6e-6	6.1e-5	7.5e-9	1.0	67	938	471
IRF5_full		1.3e-16	7.6e-5	1.5e-19	1.0	143	2232	1665
IRF9_full		1.9e-23	1.3e-4	2.2e-26	1.0	128	353	95
SPI1_full		4.4e-55	1.4e-4	5.0e-58	3.5e-6	177	1386	779
IRF4_full		9.0e-22	1.5e-4	1.0e-24	1.0	100	315	86
NFKB2_DBD		6.9e-4	1.7e-4	7.8e-7	1.0	376	2299	2302
EOMES_DBD		1.6e-5	1.7e-4	1.8e-8	1.0	152	3032	2469
MA0081.1	SPIB	1.4e-34	1.7e-4	1.5e-37	9.0e-5	134	4394	4327
Spic_DBD		7.4e-70	2.1e-4	8.3e-73	2.8e-13	177	2246	1480
MA0080.2	SPI1	1.4e-45	6.8e-4	1.6e-48	3.1e-11	170	5601	5787
MA0107.1	RELA	1.3e-4	1.2e-3	1.4e-7	1.0	283	3186	2900
TBR1_DBD		5.3e-4	2.0e-3	6.0e-7	1.0	161	3110	2603
MA0002.1	RUNX1	2.0e-8	4.1e-3	2.2e-11	1.0	176	3374	2626
TBX21_full_2		2.3e-2	4.9e-3	2.5e-5	1.0	155	2605	2129
MA0136.1	ELF5	3.8e-52	1.2e-2	4.3e-55	1.4e-17	162	5404	5624
TBX1_DBD_3		9.3e-8	3.2e-2	1.1e-10	1.0	155	2761	2450

Figure S2: **CentriMo differential enrichment.** A (partial) screenshot of the CentriMo interactive output using cancer-free TBP ChIP-seq (GM12878) regions compared with a leukaemia cell line (K562), showing all motifs with a Fisher E-value < 0.05. For each motif, the table shows its ID and name in the compendium, the local enrichment in the bound promoters ("E-value"), the differential enrichment ("Fisher E-value") in the bound vs. unbound promoters, the (unadjusted) significance of the local enrichment in the primary data set ("p-value"), the (unadjusted) significance of the local enrichment in the control data set ("Negative p-value"), the width of the region of maximum enrichment in the primary data set ("Region Width"), the number of matches in the region of maximum enrichment ("Region Matches") and finally the number of matches of the motif in the same region in the control data set ("Negative Region Matches").

Logo	Database	ID	Name	*p*-value	Adjusted *p*-value
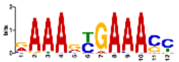	JASPAR CORE 2009 vertebrates	MA0050.1	IRF1	1.98e-198	1.75e-195
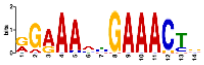	JASPAR CORE 2009 vertebrates	MA0137.1	STAT1	3.49e-171	3.09e-168
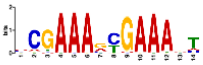	jolma2013	IRF7_DBD		1.02e-168	9.01e-166
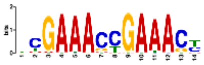	jolma2013	IRF8_DBD		2.26e-162	2.00e-159
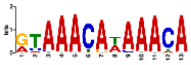	jolma2013	Foxj3_DBD_4		4.32e-153	3.82e-150
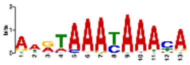	jolma2013	FOXC1_DBD		7.78e-153	6.88e-150
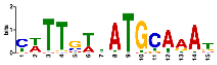	JASPAR CORE 2009 vertebrates	MA0142.1	Pou5f1	9.53e-143	8.42e-140
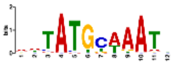	jolma2013	POU2F1_DBD		5.31e-136	4.69e-133
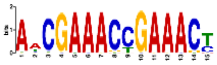	jolma2013	IRF9_full		7.79e-135	6.89e-132
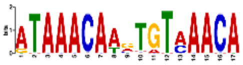	jolma2013	Foxq1_DBD		2.64e-134	2.33e-131
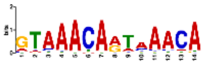	jolma2013	Foxj3_DBD_2		2.03e-133	1.80e-130
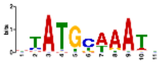	jolma2013	POU2F2_DBD		2.20e-133	1.95e-130
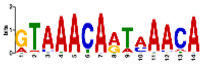	jolma2013	FOXJ2_DBD		4.27e-131	3.77e-128
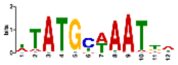	jolma2013	POU3F2_DBD_2		5.24e-131	4.63e-128
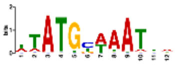	jolma2013	POU3F1_DBD		9.92e-131	8.77e-128

Figure S3: **AME differential enrichment.** A (partial) screenshot of the AME interactive output using cancer-free TBP ChIP-seq regions compared with a leukaemia cell line shows the fifteen most locally differentially enriched motifs in the JASPAR+Jolma compendium. For each motif, the table shows its logo, its database, its ID, its name in the compendium, the enrichment in the primary set versus the control set ("*p*-value"), and the Bonferroni-corrected *p*-value ("Adjusted *p*-value" – calculated as he *p*-value × number of motifs in the compendium).

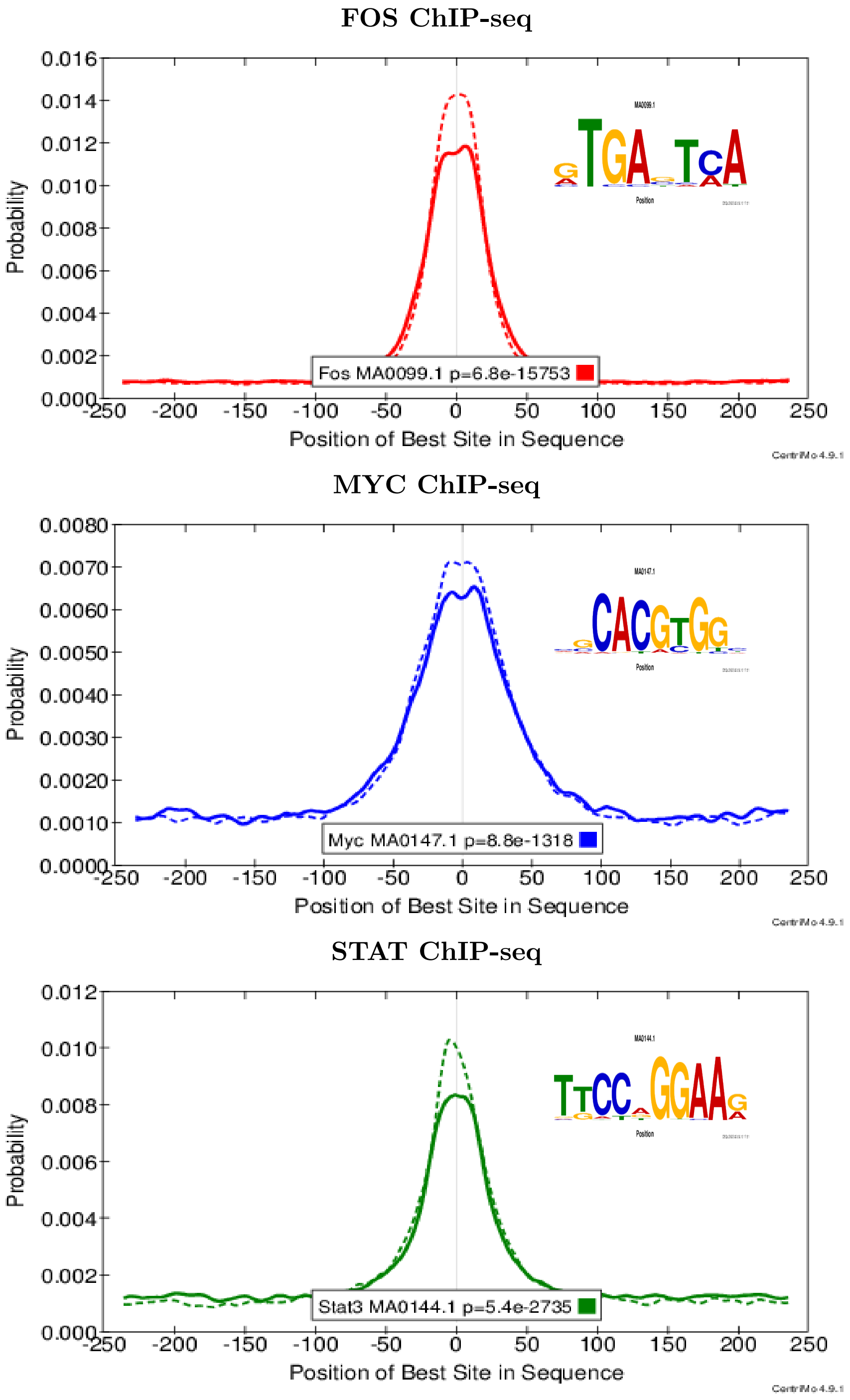

Figure S4: **The known motif for the ChIP-ed motif is more enriched in ChIP-seq peak regions from *untreated* MCF10A-ER-Src cells.** The CentriMo plots show the distribution of a known motif for the ChIP-ed TF in FOS, MYC and STAT3 ChIP-seq peak regions (top-to-bottom). Solid (dotted) curves show the positional distribution of the known motif in the tamoxifen-treated (untreated) cell ChIP-seq peak regions. Tamoxifen treatment time is 4 hours except in the STAT3 ChIP-seq experiment where it is 12 hours. JASPAR motif names and IDs and the *p*-value of the motif's central enrichment in the treated cell peaks is shown in the legend of each plot.

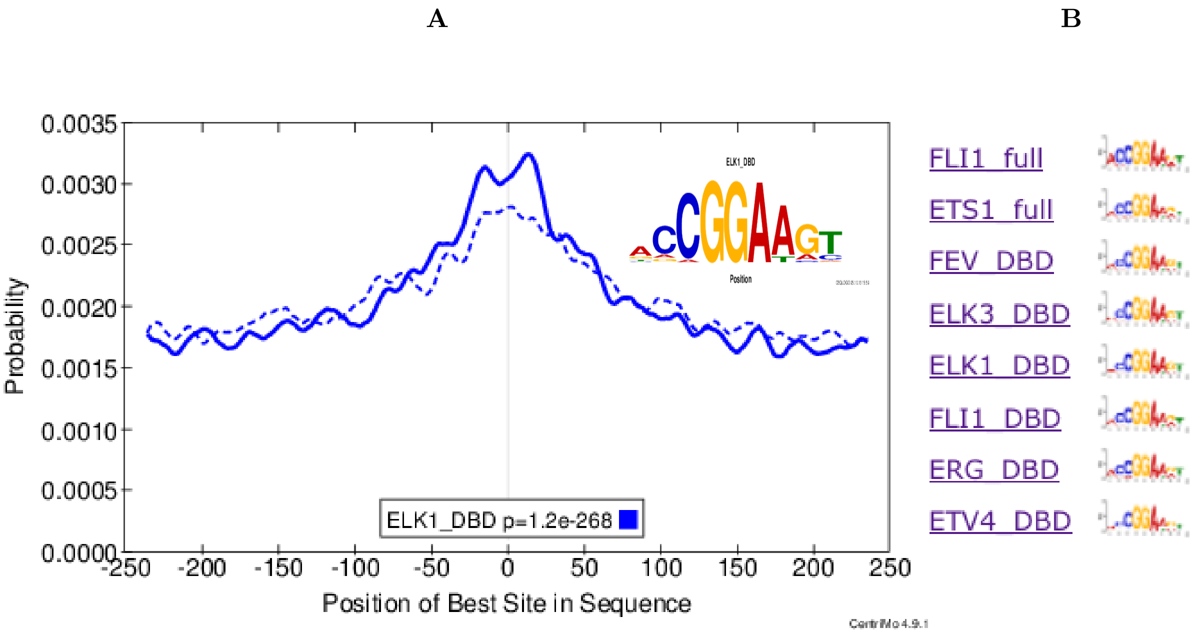

Figure S5: **ELK1 and other ETS-factor motifs are relatively enriched in FOS ChIP-seq peaks from tamoxifen treated MCF10A-ER-Src cells.** Panel A shows the central enrichment of the ELK1_DBD motif from Jolma *et al.* (2013) in the treated-cell (solid line) and untreated-cell (dotted line) FOS ChIP-seq peaks. Tamoxifen treatment was for 4 hours. Panel B shows the logos of the eight most differentially enriched ETS-factor motifs.

ID ?	Name ?	*E*-value ?	Fisher *E*-value ?	*p*-value ?	Negative *p*-value ?	Region Center ?	Region Width ?
JDP2_DBD		2.3e-20	1.4e-10	2.6e-23	1.0e0	-56	240
NFE2_DBD		1.8e-17	2.0e-10	2.0e-20	1.0	-27	224
JDP2_full		7.8e-15	3.8e-8	8.8e-18	1.0	-57.5	163
Jdp2_DBD		2.1e-16	9.4e-8	2.4e-19	1.0	-44	216
MA0089.1	NFE2L1::MafG	8.5e-19	1.2e-7	9.7e-22	2.7e-8	-22.5	126
Srebf1_DBD		6.2e-23	2.2e-5	7.0e-26	1.5e-20	-46.5	126
MA0004.1	Arnt	4.9e-20	6.6e-5	5.6e-23	5.8e-17	-34	101
ATF4_DBD		2.6e-30	9.0e-5	2.9e-33	8.3e-43	-60.5	143
DBP_full		9.5e-39	9.5e-5	1.1e-41	1.5e-73	-63.5	142
BHLHB2_DBD		3.3e-24	1.0e-4	3.7e-27	2.3e-30	-46.5	142
MA0099.2	AP1	5.5e-9	1.9e-4	6.2e-12	1.0	-68.5	163
MA0099.1	Fos	2.3e-11	2.0e-4	2.5e-14	4.4e-2	-107.5	278

Figure S6: **Local differential enrichment of motifs in FOS-bound vs. unbound promoters.** A (partial) screenshot of the CentriMo interactive output using FOS-bound and unbound promoters shows the twelve most locally differentially enriched motifs in the JASPAR+Jolma compendium. For each motif, the table shows its ID and name in the compendium, the local enrichment in the bound promoters ("*E*-value"), the differential enrichment ("Fisher *E*-value") in the bound vs. unbound promoters, the (unadjusted) significance of the local enrichment in the bound promoters ("*p*-value"), the (unadjusted) significance of the local enrichment in the unbound promoters ("Negative *p*-value"), and the coordinates of the region of maximum enrichment in the bound promoters ("Region center" and "Region width").

Table S2: **Motifs that are relatively enriched near the center of ChIP-seq peaks for the given TF in tamoxifen-treated vs. untreated MCF10A-ER-Src cells. Odds ratio is $A_{11}A_{22}/(A_{12}A_{21})$.**

FOS 4hr						
	Number of best sites near/far from ChIP-seq peak center					
	treated/near	*treated/far*	*untreated/near*	*untreated/far*		
motif	$A_{1,1}$	$A_{1,2}$	$A_{2,1}$	$A_{2,2}$	*odds ratio*	*E-value*
STAT1	11660	34770	7967	28391	1.20	5.7e-22
Stat3	12265	34638	8586	28439	1.17	7.2e-18
FLI1_full	10664	19206	7656	15808	1.15	1.3e-08
FEV_DBD	13759	26329	10024	21583	1.13	1.9e-08
ETV4_DBD	15034	31353	11009	25636	1.12	3.1e-08
ELK1_DBD	12048	22525	8770	18560	1.13	5.7e-08
FLI1_DBD	13843	26710	10132	21932	1.12	5.8e-08
ERG_DBD	14075	27339	10300	22405	1.12	8.1e-08
ELK3_DBD	12611	23697	9210	19513	1.13	8.8e-08
ETS1_full	13252	28865	9674	23623	1.12	1.1e-07

Table S3: **Motifs that are relatively enriched near the center of ChIP-seq peaks for the given TF in tamoxifen-treated vs. untreated MCF10A-ER-Src cells. Odds ratio is $A_{11}A_{22}/(A_{12}A_{21})$.**

FOS 12hr						
	Number of best sites near/far from ChIP-seq peak center					
	treated/near	*treated/far*	*untreated/near*	*untreated/far*		
motif	$A_{1,1}$	$A_{1,2}$	$A_{2,1}$	$A_{2,2}$	*odds ratio*	*E-value*
STAT1	14164	35075	9200	27173	1.19	1.6e-24
Stat3	12414	37330	8096	28922	1.19	3.3e-21
FEV	14001	26925	9258	20639	1.16	1.1e-14
ETS1_DBD	14623	27186	9858	21093	1.15	1.1e-13
ELK3_DBD	12986	25865	8696	20072	1.16	1.3e-13
FLI1_full	12281	19592	8150	15225	1.17	1.4e-13
ERG_DBD	16160	27728	10966	21536	1.14	1.6e-13
ERG_full	13737	24458	9210	18972	1.16	1.6e-13
ELK1_DBD	12405	24565	8278	19051	1.16	2.2e-13
RELA	7006	15044	4426	11595	1.22	3.3e-13

Table S4: **Motifs that are relatively enriched near the center of ChIP-seq peaks for the given TF in tamoxifen-treated vs. untreated MCF10A-ER-Src cells. Odds ratio is $A_{11}A_{22}/(A_{12}A_{21})$.**

FOS 36hr						
	Number of best sites near/far from ChIP-seq peak center					
	treated/near	*treated/far*	*untreated/near*	*untreated/far*		
motif	$A_{1,1}$	$A_{1,2}$	$A_{2,1}$	$A_{2,2}$	*odds ratio*	*E-value*
STAT1	9842	27668	8400	27918	1.18	1.5e-17
Stat3	13035	24921	11893	25125	1.10	1.4e-05
ATF4_DBD	9840	9450	8628	9454	1.14	2.2e-05
NFIL3_DBD	14236	20488	12858	20386	1.10	7.4e-05
HLF_full	11855	16945	10658	16911	1.11	0.00015
ETV1_DBD	12174	22664	11118	22783	1.10	0.00032
ETV4_DBD	12403	25233	11336	25314	1.10	0.00037
HLF	13992	23007	12676	22818	1.09	0.00048
ELK3_DBD	10189	19483	9222	19540	1.11	0.00063
ETV6_full_2	15143	22316	13980	22463	1.09	0.0011

Table S5: **Motifs that are relatively enriched near the center of ChIP-seq peaks for the given TF in tamoxifen-treated vs. untreated MCF10A-ER-Src cells. Odds ratio is $A_{11}A_{22}/(A_{12}A_{21})$.**

MYC 4hr						
	Number of best sites near/far from ChIP-seq peak center					
	treated/near	*treated/far*	*untreated/near*	*untreated/far*		
motif	$A_{1,1}$	$A_{1,2}$	$A_{2,1}$	$A_{2,2}$	*odds ratio*	*E-value*
Fos	10758	8803	12735	12529	1.20	5.7e-17
AP1	11230	10454	13905	15339	1.19	3.4e-16
STAT1	4942	10527	5773	14624	1.19	1.1e-08
Stat3	5492	10588	7013	15291	1.13	0.0027
RELA	3306	4075	4171	6071	1.18	0.0085
NF-kappaB	1609	8495	2043	13108	1.22	0.0088
NFE2L1::MafG	8949	13284	11601	18850	1.09	0.065
CEBPA	8411	12560	10428	17070	1.10	0.11
FEV	5798	6656	7161	9200	1.12	0.28
REL	7423	9907	9767	14304	1.10	0.49

Table S6: **Motifs that are relatively enriched near the center of ChIP-seq peaks for the given TF in tamoxifen-treated vs. untreated MCF10A-ER-Src cells. Odds ratio is $A_{11}A_{22}/(A_{12}A_{21})$.**

STAT3 12hr						
	Number of best sites near/far from ChIP-seq peak center					
	treated/near	*treated/far*	*untreated/near*	*untreated/far*		
motif	$A_{1,1}$	$A_{1,2}$	$A_{2,1}$	$A_{2,2}$	*odds ratio*	*E-value*
Fos	16523	13724	4353	5091	1.41	1.8e-42
AP1	16688	15934	4554	5816	1.34	9.7e-33
JDP2_DBD	12213	5656	3106	2045	1.42	1.5e-21
JDP2_full	12301	5581	3130	2011	1.42	1.1e-20
Jdp2_DBD	10888	4368	2781	1587	1.42	6.4e-17
NFE2_DBD	8330	3974	2148	1459	1.42	3e-14
NFE2L1::MafG	14052	19375	4056	6634	1.19	6.8e-09
NFE2L2	6796	8624	1918	2987	1.23	9.7e-05
MAFF_DBD	2570	2987	710	1071	1.30	0.27
Pax2	12619	20323	3791	6807	1.11	0.3

Table S7: **Motifs that are relatively enriched near the center of ChIP-seq peaks for the given TF in tamoxifen-treated vs. untreated MCF10A-ER-Src cells. Odds ratio is $A_{11}A_{22}/(A_{12}A_{21})$.**

STAT3 36hr						
	Number of best sites near/far from ChIP-seq peak center					
	treated/near	*treated/far*	*untreated/near*	*untreated/far*		
motif	$A_{1,1}$	$A_{1,2}$	$A_{2,1}$	$A_{2,2}$	*odds ratio*	*E-value*
Fos	18621	14851	4550	4894	1.35	1.7e-32
AP1	19256	17039	4875	5495	1.27	2.2e-22
JDP2_full	13842	5551	3280	1861	1.41	3.4e-20
JDP2_DBD	13987	5389	3336	1815	1.41	1.2e-19
Jdp2_DBD	12808	4177	3050	1436	1.44	5.3e-18
NFE2_DBD	9787	3557	2398	1209	1.39	1.2e-10
NFE2L1::MafG	14420	22799	3717	6973	1.19	8e-09
MEOX2_DBD	12802	20536	3320	6074	1.14	0.0077
NFE2L2	7534	9290	1986	2927	1.20	0.0078
MEOX1_full	9323	18690	2374	5451	1.15	0.097

Table S8: **Motifs that are relatively enriched near the center of ChIP-seq peaks for the given TF in tamoxifen-treated vs. untreated MCF10A-ER-Src cells but *not* enriched in untreated cells. Odds ratio is $A_{11}A_{22}/(A_{12}A_{21})$.**

FOS 4hr						
	Number of best sites near/far from ChIP-seq peak center					
	treated/near	*treated/far*	*untreated/near*	*untreated/far*		
motif	$A_{1,1}$	$A_{1,2}$	$A_{2,1}$	$A_{2,2}$	*odds ratio*	*E-value*
STAT1	11660	34770	7967	28391	1.20	5.7e-22
SPI1	7164	63276	5157	50676	1.11	0.003
CTCF	2404	18681	1752	15646	1.15	3.1
NFKB2_DBD	2020	6688	1468	5678	1.17	6.7
SPIB	25629	43024	19689	34531	1.04	27
SP1	4596	48819	3424	39177	1.08	1.6e+02
RXR::RAR_DR5	2440	22506	1810	18494	1.11	1.6e+02
INSM1	2200	20112	1630	16586	1.11	1.8e+02
NFKB1	2166	10148	1639	8562	1.11	2.4e+02
RARG_full_3	2955	24208	2230	19764	1.08	5.6e+02

Table S9: **Motifs that are relatively enriched near the center of ChIP-seq peaks for the given TF in tamoxifen-treated vs. untreated MCF10A-ER-Src cells but *not* enriched in untreated cells. Odds ratio is $A_{11}A_{22}/(A_{12}A_{21})$.**

FOS 12hr						
	Number of best sites near/far from ChIP-seq peak center					
	treated/near	*treated/far*	*untreated/near*	*untreated/far*		
motif	$A_{1,1}$	$A_{1,2}$	$A_{2,1}$	$A_{2,2}$	*odds ratio*	*E-value*
STAT1	14164	35075	9200	27173	1.19	1.6e-24
REL	19528	35862	13600	27731	1.11	2.7e-09
NF-kappaB	9659	20840	6466	16106	1.15	7.4e-09
NFKB2_DBD	2760	6957	1709	5404	1.25	2.2e-05
NFKB1	3534	10033	2275	7802	1.21	8.5e-05
ETS1	11454	65832	7880	49664	1.10	0.00057
CTCF	2644	19796	1738	15531	1.19	0.0065
SPIB	25219	47644	18112	36108	1.06	0.8
Spic_DBD	6320	9090	4413	6871	1.08	1.8e+02
SPI1	27987	46833	20423	35410	1.04	2.1e+02

Table S10: **Motifs that are relatively enriched near the center of ChIP-seq peaks for the given TF in tamoxifen-treated vs. untreated MCF10A-ER-Src cells but *not* enriched in untreated cells. Odds ratio is $A_{11}A_{22}/(A_{12}A_{21})$.**

FOS 36hr						
	Number of best sites near/far from ChIP-seq peak center					
	treated/near	*treated/far*	*untreated/near*	*untreated/far*		
motif	$A_{1,1}$	$A_{1,2}$	$A_{2,1}$	$A_{2,2}$	*odds ratio*	*E-value*
STAT1	9842	27668	8400	27918	1.18	1.5e-17
BSX_DBD	11362	36074	10396	35386	1.07	0.8
UNCX_DBD	10842	37100	9966	36309	1.06	7.3
PDX1_DBD_2	8007	32058	7281	31303	1.07	8.8
NKX6-2_full	10326	41774	9526	40831	1.06	28
HNF1A_full	2812	17982	2416	17167	1.11	45
POU6F2_full	5103	20143	4615	19727	1.08	48
Dlx2_DBD	11249	36777	10418	35961	1.06	50
Lhx8_DBD_2	9379	27558	8643	26972	1.06	50
POU4F1_DBD	5696	31440	5136	30477	1.08	58

Table S11: **Motifs that are relatively enriched near the center of ChIP-seq peaks for the given TF in tamoxifen-treated vs. untreated MCF10A-ER-Src cells but *not* enriched in untreated cells. Odds ratio is $A_{11}A_{22}/(A_{12}A_{21})$.**

MYC 4hr						
	Number of best sites near/far from ChIP-seq peak center					
motif	*treated/near* $A_{1,1}$	*treated/far* $A_{1,2}$	*untreated/near* $A_{2,1}$	*untreated/far* $A_{2,2}$	*odds ratio*	*E-value*
STAT1	4942	10527	5773	14624	1.19	1.1e-08
NF-kappaB	1609	8495	2043	13108	1.22	0.0088
NFKB1	447	4405	580	7659	1.34	1.2
NFKB1_DBD	112	771	108	1314	1.77	9.7
NFKB2_DBD	338	3035	412	5023	1.36	9.7
EN1_full_2	8278	9742	9870	12556	1.08	12
NR2E1_full	8835	10246	11001	13737	1.08	15
TEAD1_full	7668	13134	9882	18167	1.07	23
MEOX2_DBD	10652	8987	13226	11957	1.07	33
MZF1_1-4	1959	19943	2478	28273	1.12	36

Table S12: **Motifs that are relatively enriched near the center of ChIP-seq peaks for the given TF in tamoxifen-treated vs. untreated MCF10A-ER-Src cells but *not* enriched in untreated cells. Odds ratio is $A_{11}A_{22}/(A_{12}A_{21})$.**

STAT3 12hr						
	Number of best sites near/far from ChIP-seq peak center					
motif	*treated/near* $A_{1,1}$	*treated/far* $A_{1,2}$	*untreated/near* $A_{2,1}$	*untreated/far* $A_{2,2}$	*odds ratio*	*E-value*
Hoxa2_DBD	9833	18151	2888	5845	1.10	42
SPIB_DBD	3460	7705	982	2541	1.16	53
POU1F1_DBD	11408	15027	3317	4723	1.08	2.4e+02
VAX2_DBD	10985	18688	3275	6000	1.08	2.7e+02
En2_DBD	11918	11245	3591	3666	1.08	3.1e+02
MZF1_1-4	2920	29401	856	9662	1.12	4e+02
ZIC3_full	1411	6639	478	2643	1.18	4.2e+02
Nkx6-1_DBD	15217	16557	4643	5387	1.07	4.2e+02
HMBOX1_DBD	9710	15903	2946	5192	1.08	4.4e+02
HOXA2_DBD	10654	18953	3213	6110	1.07	5.4e+02

Table S13: **Motifs that are relatively enriched near the center of ChIP-seq peaks for the given TF in tamoxifen-treated vs. untreated MCF10A-ER-Src cells but *not* enriched in untreated cells. Odds ratio is $A_{11}A_{22}/(A_{12}A_{21})$.**

STAT3 36hr						
	Number of best sites near/far from ChIP-seq peak center					
motif	*treated/near* $A_{1,1}$	*treated/far* $A_{1,2}$	*untreated/near* $A_{2,1}$	*untreated/far* $A_{2,2}$	*odds ratio*	*E-value*
MEOX2_DBD	12802	20536	3320	6074	1.14	0.0077
DLX5_FL	4296	29489	1025	8412	1.20	0.11
En2_DBD	8210	17676	2084	5167	1.15	0.13
POU1F1_DBD	11256	18210	2834	5193	1.13	0.22
Meox2_DBD	8462	24168	2135	6918	1.13	0.57
Hoxa2_DBD	7162	24178	1794	6926	1.14	0.7
VAX1_DBD	7031	26077	1767	7449	1.14	1.6
EMX2_DBD	5422	19911	1340	5681	1.15	2.4
HOXB5_DBD	6806	30355	1739	8783	1.13	2.5
EVX2_DBD	6883	24577	1756	7086	1.13	4.5

References

ENCODE Consortium (2012). An integrated encyclopedia of DNA elements in the human genome. *Nature*, **489**(7414), 57–74.

Greiner, J., Ringhoffer, M., Taniguchi, M., Hauser, T., Schmitt, A., Döhner, H., and Schmitt, M. (2003). Characterization of several leukemia-associated antigens inducing humoral immune responses in acute and chronic myeloid leukemia. *International Journal of Cancer*, **106**(2), 224–231.

Jolma, A., Yan, J., Whitington, T., Toivonen, J., Nitta, K. R., Rastas, P., Morgunova, E., Enge, M., Taipale, M., Wei, G., Palin, K., Vaquerizas, J. M., Vincentelli, R., Luscombe, N. M., Hughes, T. R., Lemaire, P., Ukkonen, E., Kivioja, T., and Taipale, J. (2013). DNA-binding specificities of human transcription factors. *Cell*, **152**(1-2), 327–339.

McLeay, R. C. and Bailey, T. L. (2010). Motif Enrichment Analysis: a unified framework and an evaluation on ChIP data. *BMC Bioinformatics*, **11**, 165.

van der Velden, V. H. J., Hochhaus, A., Cazzaniga, G., Szczepanski, T., Gabert, J., and van Dongen, J. J. M. (2003). Detection of minimal residual disease in hematologic malignancies by real-time quantitative pcr: principles, approaches, and laboratory aspects. *Leukemia*, **17**(6), 1013–1034.
